# U.S. Funding Cuts and Health System Challenges in Community-Based HIV Programmes in Rural KwaZulu-Natal, South Africa: A Qualitative Study

**DOI:** 10.3390/ijerph23080951

**Published:** 2026-07-24

**Authors:** Siphelele Thamsanqa Ngubane, Dumile Gumede

**Affiliations:** Faculty of Health Sciences, Durban University of Technology, Durban 4001, South Africa; 22494841@dut4life.ac.za

**Keywords:** HIV, funding instability, health systems, community-based programmes, South Africa, health workforce, service delivery, WHO building blocks, 95–95–95

## Abstract

**Highlights:**

**Public health relevance—How does this work relate to a public health issue?**

- Sustaining community-based HIV prevention and support services is a major public health issue in high-burden rural settings because outreach, tracing, counselling, and linkage to care are essential for reaching underserved populations.
- The study is relevant to health system resilience, showing how abrupt donor funding shocks can weaken the functions needed to achieve the 95–95–95 targets, particularly testing access, treatment linkage, retention, and continuity of care.

**Public health significance—Why is this work of significance to public health?**

- This study is significant because it shows that funding instability affects more than programme budgets; it disrupts service delivery, workforce capacity, information systems, prevention commodities, financing, and governance, with consequences for the broader HIV response.
- It is also significant because it demonstrates that community-based HIV programmes are part of the local health system infrastructure, meaning that funding cuts can undermine progress toward the second 95 (treatment coverage) and third 95 (viral suppression).

**Public health implications—What are the key implications or messages for practitioners, policymakers and/or researchers in public health?**

- Policymakers and donors should protect community-based HIV functions during funding transitions.
- Stronger domestic financing, transition planning, and integration between community programmes and public-sector services are needed to safeguard progress toward 95–95–95 and to prevent donor shocks from reversing HIV gains in high-burden rural settings.

**Abstract:**

Community-based organisations (CBOs) play a critical role in implementing HIV programmes, many of which have historically relied on United States (U.S.) donor funding. Recent U.S. funding cuts have disrupted community-based HIV programmes, underscoring the need to understand implementers’ experiences to support sustainable service delivery. This study explored how funding instability affected community-based HIV programmes in rural KwaZulu-Natal, South Africa. A qualitative, exploratory design was employed using interviews and focus group discussions with purposively selected youth frontline workers and programme managers (*n* = 26). The Health Systems Building Blocks framework guided thematic analysis. Participants described disruptions in outreach and prevention services, contract terminations and reduced working hours among frontline personnel, weakened data and follow-up systems, shortages or reduced local availability of HIV prevention commodities, lack of transition planning, and abrupt program closure without sustainability measures. U.S. funding cuts disrupted essential community-based HIV services in rural KwaZulu-Natal, exposing vulnerabilities in prevention, outreach, workforce, and monitoring systems. Sustainable financing and stronger integration of community organisations into health systems are needed to protect HIV service continuity.

## 1. Introduction

South Africa has the largest HIV epidemic in the world, with 7.8 million people living with HIV [1]. However, the country has made substantial progress in treatment coverage, viral suppression, and prevention over the past two decades [2]. However, these gains have depended on a mixed financing architecture in which domestic resources support core treatment procurement. In contrast, donor funding, particularly through the United States (U.S.) President’s Emergency Plan for AIDS Relief (PEPFAR), USAID, CDC and associated implementing partners, have supported critical complementary functions, including community outreach, tracing, counselling, key-population programming, monitoring systems, and workforce supplementation [3,4]. In 2023, the most recent year with complete expenditure data, PEPFAR contributed 21% of South Africa’s HIV spending, amounting to part of the country’s total HIV budget of $1.86 billion, while the Global Fund provided a further 3% [5]. However, donor and domestic contributions were not evenly spread across programme areas. Although the South African government financed most antiretroviral treatment (86%), PEPFAR played a much larger role in prevention, covering 50% of prevention-related expenditure, including major support for voluntary medical male circumcision (VMMC) and pre-exposure prophylaxis (PrEP) [5]. PEPFAR also funded important treatment retention activities delivered by community-based health workers, services for key populations, and broader health system strengthening efforts, including logistics, supply chain support, and data systems [4,5].

Beginning in early 2025, the abrupt suspension and termination of many U.S.-funded agreements in South Africa generated widespread concern about the continuity of HIV services. UNAIDS reported that tens of thousands of HIV response roles were affected across national and priority district systems, with disruptions in testing, outreach, PrEP services, mobile programs, data systems, and community-led monitoring [1]. By March 2025, service interruptions were already being documented nationally, while later evidence from KwaZulu-Natal showed that a large proportion of facilities experienced staffing, operational, and service disruptions linked to funding instability [6].

The relevance of these disruptions is underscored by the UNAIDS 95–95–95 targets, which call for 95% of people living with HIV to know their status, 95% of those diagnosed to receive sustained antiretroviral therapy (ART), and 95% of those on treatment to achieve viral suppression by 2025 [7]. In South Africa, the urgency of these targets was reflected in the February 2025 launch of the national “Close the Gap” campaign, which sought to identify and re-engage 1.1 million people living with HIV who knew their status but were not yet on treatment, highlighting that the largest gap in the HIV response lies in the second 95 [8]. In this context, disruptions to community-based HIV programmes are highly consequential because they weaken the outreach, linkage, adherence support, and follow-up systems that are essential for progress across all three stages of the cascade [9].

This policy context matters because community-based HIV programmes contribute directly to all three stages of the HIV cascade [10]. Community outreach and testing support strengthen the first 95; tracing, counselling, linkage, and treatment initiation support strengthen the second 95; and adherence support, follow-up, and community retention mechanisms strengthen the third 95 by helping people remain in care long enough to achieve viral suppression [9]. Funding instability that weakens these community-based functions, therefore, threatens more than programme administration; it threatens progress toward epidemic control.

These developments matter particularly in rural KwaZulu-Natal, where community-based HIV programs are often central to reaching young people, key populations, and other underserved groups who face barriers to routine facility care [3]. Recent studies from rural KwaZulu-Natal show that peer navigators, mobile services, and youth-centred community programs improve HIV testing uptake, linkage to care, ART initiation, and access to prevention by reducing the social and geographic distance between people and formal health services [11,12,13]. In settings where stigma, transport costs, and facility-level barriers remain significant [13], the contraction of community programs may therefore produce effects that extend far beyond non-government organisation (NGO) operations alone.

Although modelling and surveillance reports are beginning to quantify the effects of the 2025 funding disruption, less is known about how these cuts were experienced by those working within community-based HIV programs and how such instability affected the functioning of local health systems at the service-delivery level. This manuscript addresses that gap by drawing on qualitative findings from rural KwaZulu-Natal to examine the health system consequences of funding instability for community-based HIV programming. It asks: How did U.S. funding cuts affect the functioning of community-based HIV programmes in rural KwaZulu-Natal, and what do these effects reveal about the role of such programmes within the broader health system and South Africa’s progress toward 95–95–95?

This manuscript is guided by the World Health Organisation (WHO) Health Systems Building Blocks framework, which conceptualises health systems as six core components: service delivery, health workforce, health information systems, access to essential medical products and technologies, financing, and leadership and governance [14]. The framework is useful for examining how shocks affect the functioning of a health system, not only through direct resource loss but also through disruptions to the interdependence among these components [15]. WHO’s framing emphasises that health systems are not defined solely by commodities or facilities, but by the interactions among people, processes, financing arrangements, infrastructure, information, and governance [16].

## 2. Methods

This manuscript draws on primary qualitative data from a broader study exploring the experiences of youth frontline workers and other stakeholders involved in delivering HIV prevention programmes for young people in rural KwaZulu-Natal, South Africa. The broader study used an exploratory qualitative design and included in-depth interviews, focus group discussions, and key informant interviews. In the current manuscript, we conducted a focused thematic analysis of the primary qualitative data on funding instability and its effects on community-based HIV programmes and the wider health system.

The study was conducted in the uMkhanyakude district, KwaZulu-Natal, a province with a high HIV burden and a long history of donor-supported and community-based HIV programming. The setting was purposively selected because organisations in the area were implementing ongoing community-based HIV programmes targeting young people [17,18].

The study used purposive sampling to recruit 26 participants from two CBOs implementing HIV programmes in the study setting. Participants included youth frontline workers involved in community-based HIV service delivery, and programme managers responsible for implementation and service coordination.

Data were generated through multiple qualitative methods, including in-depth interviews with youth frontline workers (*n* = 13), two focus group discussions with youth frontline workers (*n* = 9), and key informant interviews with programme managers (*n* = 4). All interviews and focus group discussions were conducted in isiZulu at the participating CBO premises, lasted 45–60 min, and were audio-recorded with participants’ consent. Data collection occurred from August to October 2025, during a period when U.S. funding cuts to HIV-related programmes had recently been announced. Interview and discussion guides explored participants’ roles in programme implementation, their experiences of delivering HIV services, perceived barriers and facilitators to service delivery, and their views on changes linked to financial instability. Field observations undertaken during recruitment and site engagement further enriched the dataset by documenting workforce contraction, reduced programme activity, and organisational instability.

All audio-recorded interviews and focus group discussions were transcribed verbatim and analysed using thematic analysis [19]. The analysis involved repeated reading of the transcripts to become familiar with the data, followed by coding meaningful segments and grouping related codes into broader themes and sub-themes. A focused thematic analysis was undertaken using the WHO Health Systems Building Blocks framework to examine how funding instability affected service delivery, the health workforce, information systems, access to essential medicines and technologies, financing, and leadership and governance [14]. Field observation notes were used to complement and contextualise the interview and focus group data.

Trustworthiness was enhanced by attending to credibility, dependability, confirmability, and transferability [20]. Credibility was strengthened through multiple qualitative methods and participant groups, enabling triangulation of perspectives. Dependability was supported by a systematic, iterative analytic process that included verbatim transcription and structured coding of the data. Confirmability was enhanced with field notes to contextualise interpretations and maintain closeness to participants’ accounts. Transferability was supported by a clear description of the study setting, participants, and data collection procedures, enabling readers to assess the relevance of the findings to similar contexts.

Ethical approval for the parent study was obtained from the Institutional Research Ethics Committee [Ethics Clearance Number: IREC 139/25]. Permission to conduct the study was also obtained from the participating CBOs. All participants were informed about the purpose of the study, the voluntary nature of participation, and their right to withdraw at any stage without consequence. Written informed consent was obtained before data collection, and all interviews and focus group discussions were audio-recorded only with participants’ permission. To protect confidentiality, transcripts were anonymised, and participants are identified in this manuscript using unique study codes.

## 3. Findings

This section presents the findings on how recent U.S. funding cuts affected community-based HIV programmes in rural KwaZulu-Natal. The findings are organised using the WHO Health Systems Building Blocks framework to illustrate how funding instability affected distinct yet interconnected dimensions of the local HIV response.

### 3.1. WHO Health System Building Blocks

The findings show that funding instability affected community-based HIV programmes across all six WHO health system building blocks. Rather than functioning as a single financial constraint, the U.S. funding cuts produced linked disruptions across service delivery, workforce capacity, information systems, commodities and technologies, governance arrangements, and programme continuity.

#### 3.1.1. Service Delivery Disruptions

Participants consistently reported a decline in outreach capacity and service continuity following funding cuts. Community activities were paused, scaled back, or stopped entirely, and some organisations shifted from community-based service delivery to more restricted clinic-based work. One participant explained,

*“There is nothing that we are doing right now as an organisation until we get more funding. Since there are budget cuts, we are no longer going to communities.”*

(KII-3, programme manager)

Another stated,

*“We have stopped all our outreach programmes; we only work in clinics to provide counselling because there is no funding to continue with these programmes.”*

(IDI-1, youth)

Participants also expressed concern that the suspension of programs left young people without trusted access points for prevention and support:

*“It is disheartening to see that we have been supporting young people while the programme was still running, but now that it has come to an end, some are left without assistance. For example, some young people obtained their ART, condoms, and lubricants from us rather than from the clinic, and when funding is lost, the programme ends, and many of them are left without support.”*

(KII-1, programme manager)

Another one stated,

*“After the funding ended, the programme stopped, and the people we had supported were left without help, making it seem as though our work as an organisation had been in vain.”*

(FGD-2, youth)

These findings suggest that community-based services were not peripheral add-ons, but critical components of local HIV delivery systems. When funding instability reduced outreach, access narrowed, especially for young people who did not routinely use clinic-based services.

#### 3.1.2. Health Workforce Instability

Participants reported widespread workforce instability following funding cuts, including job losses, contract non-renewals, salary reductions, and reduced working days. Some staff had already lost their jobs by the time data collection began, while others had moved from full-time to part-time work. One participant explained:

*“Some staff members had their contracts expire, and they were not renewed, and some of them were forced to take a salary cut while working from home.”*

(IDI-6, youth)

Another explained:

*“Our organisation hires more youth workers, but they are not here today… since the organisation is facing financial challenges due to budget cuts, they are only here two or three days a week.”*

(KII-3, programme manager)

Participants emphasised that staff instability weakened continuity of care because youth frontline workers often built ongoing trust-based relationships with beneficiaries. Where workers lost employment, follow-up and relational continuity were lost.

#### 3.1.3. Health Information and Follow-Up Systems

Although participants did not always explicitly name information systems, their accounts showed that follow-up, tracing, record flow, and continuity mechanisms were weakened when funding instability reduced staff and program activity. Participants described losing contact with beneficiaries, interruptions in program monitoring, and weakened ability to sustain engagement after programme closure. One participant reflected:

*“I lost contact with some youth beneficiaries who had been enrolled in our HIV prevention programmes because budget cuts resulted in the non-renewal of youth frontline workers’ contracts.”*

(KII-4, programme manager)

Others indicated that community-linked support systems had effectively served as informal continuity mechanisms for young people reluctant to collect treatment or prevention commodities from clinics.

These findings suggest that information and continuity systems in community-based HIV programs were embedded in people and relationships rather than solely in formal databases.

#### 3.1.4. Medicines, Products and Technologies

Participants reported shortages of condoms, HIV testing kits, pamphlets, and PrEP availability at community sites, which directly affected service delivery. In some cases, youth frontline workers had to borrow materials from nearby health facilities to keep functioning, as indicated below:

*“With the recent funding cuts, we don’t have enough condoms, pamphlets, PrEP and HIV testing kits.”*

(IDI-12, youth)

*“We didn’t have HIV testing kits on site, so we ended up borrowing from nearby health facilities, and we were able to continue working.”*

(IDI-2, youth)

Others highlighted the problem of promoting services that were not locally available:

*“If we speak to young people about PrEP, we should focus on something that is actually in stock, rather than discussing products that are not available on site.”*

(IDI-4, youth)

These accounts indicate that the cuts not only reduced financing but also weakened the practical availability and credibility of community-based HIV prevention.

#### 3.1.5. Financing and Sustainability

Participants understood funding instability not simply as a temporary shortfall, but as a threat to the very existence of community-based HIV programmes. They criticised short-term programmes that ended without transition plans and described sustainability as one of the most serious weaknesses in implementation. One participant said:

*“The programmes should not be like fly-by-night, whereby when it’s over, it just over like that.”*

(IDI-4, youth)

A programme manager explained:

*“The problem with our programme is sustainability. We have started the programme, but due to budget cuts, the programme ends, and we must leave what we have started without seeing it to the end.”*

(KII-2, programme manager)

Participants were especially concerned that programmes ended while beneficiaries still had ongoing needs and queries.

#### 3.1.6. Leadership and Governance Gaps

Participants identified significant governance weaknesses in the management of funding contractions. These included weak strategic planning for sustainability, abrupt programme closure, poor handover processes, and uneven geographic coverage of services. Relationship disruption was a recurring theme: participants argued that communities, especially children and vulnerable youth, lost trusted service providers and health professionals when organisations’ funding ended. One participant explained the consequences of abrupt programme closure:

*“When programmes end, or staff lose their jobs, new social workers may be introduced, creating a continuity gap that makes it difficult for children to build trust and open up to someone new.”*

(KII-3, programme manager)

Similarly, another participant highlighted problems with weak transition plans for sustainability:

*“If such programmes are introduced, there should be a clear commitment to see them through to completion, rather than ending them halfway while people are still seeking support.”*

(FGD-1, youth)

Together, these findings show that funding instability exposed not only resource dependence, but also governance weaknesses in transition planning, accountability, and long-term programme stewardship.

## 4. Discussion

This study found that abrupt U.S. funding cuts did not simply reduce the financial resources available to community-based HIV programmes in rural KwaZulu-Natal; they disrupted the social, organisational, and operational infrastructure through which HIV services reach communities. Although the analysis was informed by the WHO Health Systems Building Blocks framework, the central contribution of this study is not only that each building block was affected. Rather, the findings show that donor-supported community-based organisations perform cross-cutting health system functions that are essential to prevention, linkage to care, retention, monitoring, and service responsiveness [14,21]. The key implication is that CBOs are not peripheral to the HIV response. They are integral to the health system, especially in high-burden rural settings where public clinics alone cannot fully meet the needs of young people, key populations, and communities facing barriers to care.

The discussion, therefore, focuses on five higher-level implications of the findings. First, community-based organisations should be understood as essential components of the health system. Second, domestic financing of antiretroviral medicines does not fully protect the HIV response from external funding shocks. Third, abrupt funding cuts weaken the prevention, linkage, retention, and data functions that sustain progress toward HIV targets. Fourth, health system resilience requires stronger governance, contingency financing, and transition planning. Fifth, sustainable community-based HIV programming requires diversified and institutionalised support, including but not limited to government financing, local partnerships, and community participation.

### 4.1. Community-Based Organisations Are Essential Health System Infrastructure, Not Marginal Implementers

A major implication of this study is that CBOs should not be treated as temporary, external, or supplementary actors in the HIV response. Participants’ accounts showed that these organisations perform functions that formal clinic-based services often struggle to provide consistently, including outreach, youth-friendly engagement, psychosocial support, counselling, tracing, prevention education, linkage to care, and follow-up for people at risk of disengagement. This is particularly important in rural KwaZulu-Natal, where transport costs, stigma, long waiting times, fear of disclosure, and limited youth-friendly services may reduce the accessibility and acceptability of facility-based care [11,12,17,18].

The funding cuts, therefore, weakened not only individual projects but also the community-facing infrastructure that enables people to access and remain connected to HIV services. This finding aligns with emerging work on community health systems, which argues that community actors, data flows, supervision structures, and local delivery platforms should be recognised as part of health system functioning rather than as informal extensions of formal services [21]. It also supports UNAIDS guidance emphasising the importance of community-led responses in reaching populations that formal health services may underserve [22].

For policymakers and programme planners, the key message is that community-based HIV programmes should be planned, financed, monitored, and protected within the health system itself. Treating these organisations as donor-funded add-ons creates vulnerability because essential service functions can disappear when external financing is withdrawn. Health systems strengthening should therefore explicitly include community-based delivery platforms, peer navigators, counsellors, outreach workers, community data systems, and civil society monitoring mechanisms within national, provincial, and district health planning.

### 4.2. Domestic ART Financing Does Not Fully Protect the HIV Response from External Funding Shocks

A second implication is that South Africa’s relatively strong domestic financing of antiretroviral medicines does not fully insulate the HIV response from the effects of donor withdrawal. South Africa funds a substantial proportion of its HIV treatment programme, and government-financed antiretroviral medicines remained central to the national response during the funding disruption [5,23]. However, participants did not primarily describe a collapse in government-funded ART procurement. Instead, they described losing the people, systems, commodities, and community platforms that help individuals access testing, initiate treatment, remain adherent, return to care after interruption, and access prevention services.

This distinction is important. A country may finance most of its medicines domestically while still depending on external funding for strategic and labour-intensive functions such as community outreach, differentiated service delivery, tracing, counselling, demand creation for prevention, data capture, programme monitoring, and services for key and vulnerable populations [1,3,4,6,24]. These functions may represent a smaller share of total HIV expenditure, but their removal can have disproportionate effects because they support the practical operation of the HIV cascade.

This study, therefore, cautions against equating domestic ART procurement with full HIV programme resilience. The resilience of the HIV response should be judged not only by whether medicines remain available, but also by whether the broader service ecosystem that enables people to access and remain in care is sustained. For policymakers, health financing assessments should map not only which commodities are domestically funded, but also which essential service delivery, workforce, monitoring, prevention, and community engagement functions remain donor dependent. Without such mapping, funding shocks may appear financially limited while producing wide operational consequences.

### 4.3. Funding Cuts Weaken the Prevention, Linkage, Retention, and Data Functions That Sustain the HIV Cascade

A third implication is that abrupt funding cuts are likely to have their most serious effects at points in the HIV cascade that depend on active community engagement. Participants described disruptions to outreach, HIV testing, prevention education, condom distribution, PrEP access, counselling, tracing, and follow-up. These are precisely the functions that help identify people at risk, link them to services, support treatment initiation, prevent disengagement, and re-engage those who have fallen out of care. Evidence from South Africa and other settings similarly shows that PEPFAR- and USAID-linked disruptions affected HIV testing, tracing, outreach, mobile services, drop-in centres, community monitoring, and key population programmes [1,6,24,25].

This has particular significance for young people and rural communities. Community-based and mobile models can reduce the geographic, social, and symbolic distance between young people and formal health services [12]. Previous work in rural KwaZulu-Natal has also shown that adolescent and youth engagement with HIV prevention programmes is shaped by social context, caregiver relationships, stigma, and the accessibility of services [17,18]. In such contexts, referral to a public clinic is not always an adequate substitute for trusted community-based access points. When community programmes contract, those most affected may be the very groups least able or willing to navigate standard facility-based systems.

The findings also show that data and monitoring functions should not be understood as merely administrative. Community-based data systems support tracing, follow-up, outreach planning, hotspot identification, stock visibility, programme accountability, and early identification of service gaps [6,11]. When funding cuts weaken data capture, reporting, and community-led monitoring, the health system loses part of its ability to identify where people are being missed and to respond in real time. This threatens progress toward the 95–95–95 targets because testing, treatment initiation, retention, and viral suppression depend on continuous identification and follow-up of people across the cascade [7,9,10].

The health consequences of these disruptions could be substantial, but they cannot be directly estimated from the present qualitative data. Modelling studies suggest that cessation of PEPFAR-supported services could reverse gains in HIV incidence and mortality if government and other actors do not rapidly replace affected functions [5,26]. However, this study did not include routine service utilisation, mortality, hospitalisation, or longitudinal cascade data. The findings, therefore, identify plausible pathways of risk rather than providing numerical estimates of additional infections, hospitalisations, or deaths. Future research should combine qualitative evidence with routine programme data and modelling to estimate these downstream health consequences more directly.

### 4.4. Health System Resilience Requires Contingency Financing, Transition Planning, and Stronger Governance

A fourth implication concerns governance and resilience. Participants’ accounts suggest that the effects of the funding cuts were intensified by their abruptness and by limited transition planning. Organisations had little time to retain staff, complete activities, communicate with beneficiaries, transfer responsibilities, or secure alternative funding. As a result, the shock was transferred downward to community-based organisations, frontline workers, and service users.

This finding is consistent with the resilience literature in health systems, which defines resilience as the capacity to absorb, adapt to, and transform in response to shocks while maintaining essential functions [15]. In this study, maintaining ART procurement was important but insufficient. Resilience also required preserving community outreach, tracing, prevention, counselling, data, and linkage-to-care functions. Weak transition planning and governance meant that these functions were not adequately protected when external funding was disrupted [15,16].

For donors, the findings highlight the need for responsible transition planning, including phased handover, transparent timelines, and joint planning with government, implementing partners, civil society, and communities. Global sustainability and transition guidance similarly warns that abrupt withdrawal of external support can reverse health gains and therefore requires planned transition, domestic resource mobilisation, and alignment with national systems [27].

For government, the findings point to the need for routine mapping of donor-supported functions and contingency financing mechanisms that protect not only medicines, but also the workforce, data systems, outreach platforms, and prevention services that enable HIV programmes to function effectively [14,27].

### 4.5. Sustainable Community-Based HIV Programming Requires Diversified and Institutionalised Support

A fifth implication is that sustainability will require diversified and institutionalised support for community-based HIV programmes. Participants’ experiences show the risks of relying heavily on a single external funding source for essential community health functions. Sustainable models should combine stronger domestic public financing, integration of community-based organisations into district health planning, local partnerships, philanthropic support, and meaningful community participation [14,15,22,27].

Government funding remains central. If community-based organisations perform core health system functions, including outreach, counselling, tracing, data support, prevention education, and linkage to care, these roles should be reflected in public budgeting and contracting mechanisms. This is consistent with guidance on sustaining community-led HIV responses and strengthening transition planning when external financing declines [22,27].

Local businesses and philanthropic actors may also support sustainability through corporate social responsibility initiatives focused on youth health, HIV prevention, transport support, digital tools, and community wellness. However, such partnerships should complement, not replace, government responsibility for essential health services [22,27].

Volunteerism can strengthen community ownership and trust, but it should not substitute for skilled and remunerated health system work. Functions such as counselling, PrEP support, tracing, data management, and linkage to care require training, supervision, confidentiality, and continuity. WHO guidance therefore supports formal health system support and appropriate remuneration for community health workers [28].

### 4.6. Study Limitations

This study has several limitations that should be considered when interpreting the findings. First, because data collection occurred amid rapidly evolving programme uncertainty, participants’ accounts may have been influenced by immediate concerns about job insecurity, organisational instability, and possible programme closure, potentially amplifying perceptions of disruption and risk while also reflecting the lived reality of the funding shock. Second, the WHO Health Systems Building Blocks framework was used as an interpretive lens rather than as the basis for a full health systems assessment. The study, therefore, explains how participants experienced and understood the effects of funding cuts, but it does not quantify the magnitude of disruption across the entire provincial or national HIV response. Third, this study did not include routine service utilisation, financial, hospitalisation, mortality, or longitudinal HIV cascade data. As a result, it cannot estimate the number of additional HIV infections, treatment interruptions, hospitalisations, or deaths attributable to the funding cuts. Instead, the findings identify plausible pathways through which disruptions to outreach, prevention, linkage, tracing, data systems, and support for retention may affect HIV outcomes. Finally, the study did not directly measure HIV cascade outcomes, but instead inferred implications for the 95–95–95 targets from disruptions in outreach, linkage, follow-up, and adherence support.

### 4.7. Recommendations

The findings point to three key recommendations to strengthen health systems and protect HIV service continuity during donor transitions.

First, government and donors should jointly conduct a comprehensive mapping of donor-supported HIV functions and develop contingency financing mechanisms to protect essential services during funding shocks. This mapping should include outreach, prevention, tracing, counselling, data systems, support for key and vulnerable populations, HIV testing, linkage to care, treatment continuity, and re-engagement of people who have disengaged from care [5,14,15,22,27]. Identifying which services are most at risk during funding withdrawal would enable government and partners to prioritise critical community-based HIV services and prevent disruptions. Global transition guidance emphasises that abrupt withdrawal of external support can threaten service continuity and reverse health gains [27].

Second, CBOs should be formally integrated into district, provincial, and national health system planning and financing structures. This should include clearer referral pathways, shared data systems, formal recognition of CBOs as part of the health system’s delivery infrastructure, and inclusion of community-led HIV responses in costing and budgeting processes [21,22]. Sustainable financing should combine public funding, responsible donor transition planning, local partnerships, corporate social responsibility initiatives, and community participation. However, volunteerism should not replace paid and supervised roles that require specialised skills, confidentiality, continuity, and accountability. WHO guidance supports formal training, supervision, integration, and remuneration for community health workers [28].

Third, future research and regional collaboration should strengthen evidence on the health impacts of funding disruptions and support cross-country learning on sustainable HIV service delivery. Future studies should quantify the consequences of funding disruptions on HIV testing, ART initiation, retention in care, viral suppression, hospitalisations, mortality, and new infections [5,25,26,29,30]. Regional institutions such as the Africa CDC could support shared learning on donor transition, domestic financing, workforce protection, and continuity of essential HIV services. Africa CDC’s Strategic Plan highlights regional coordination, resilient health systems, sustainable financing, and workforce strengthening as key priorities [30].

## 5. Conclusions

This study shows that U.S. funding cuts operated as a systemic stressor for community-based HIV programmes in rural KwaZulu-Natal. The consequences were not limited to reduced budgets or suspended projects. Rather, the cuts weakened the community-facing infrastructure that supports HIV prevention, linkage to care, treatment continuity, tracing, counselling, data use, and engagement with young people and other underserved groups. The central implication is that CBOs are not marginal to the HIV response; they should be recognised as integral components of the health system. South Africa’s domestic financing of antiretroviral medicines is an important strength, but it does not fully protect the HIV response when donor funding supports the workforce, outreach, prevention, data, and relational systems that enable people to access and remain in care. Strengthening resilience will require more than replacing lost donor funds. It will require deliberate transition planning, contingency financing, stronger governance of donor-supported functions, sustainable investment in community-based delivery, and regional learning on how African health systems can protect essential services during external financing shocks. Future research should complement qualitative evidence with quantitative estimates of the health consequences of programme disruption, including effects on HIV incidence, hospitalisation, mortality, and progress toward the 95–95–95 targets.

## Data Availability

The data presented in this study are not publicly available due to ethical and confidentiality considerations. Data may be made available from the corresponding author upon reasonable request, subject to approval by the relevant ethics committee.

## Abbreviations

The following abbreviations are used in this manuscript:

CBO	Community-based organisation
US	United States
PEPFAR	President’s Emergency Plan for AIDS Relief
WHO	World Health Organisation
HIV	Human Immunodeficiency Virus
ART	Antiretroviral Therapy
ARV	Antiretroviral
PREP	Pre-exposure prophylaxis
UNAIDS	Joint United Nations Programme on HIV/AIDS
NGO	Non-government organisation
USAID	United States Agency for International Development
CDC	Centres for Disease Control and Prevention
VMMC	Voluntary medical male circumcision
LGBTQI+	Lesbian, Gay, Bisexual, Queer/Questioning, Transgender, Intersex, and others
IDI	In-depth interview
KII	Key informant interview
FGD	Focus group discussion
